# Acute HIV infection with presentations mimicking acalculous cholecystitis: A case report

**DOI:** 10.1097/MD.0000000000026653

**Published:** 2021-07-16

**Authors:** Wang-Da Liu, Chih-Ning Cheng, Ya-Ting Lin, Ching-Hua Kuo, Shu-Yuan Ho, Chien-Ching Hung

**Affiliations:** a Department of Internal Medicine, National Taiwan University Hospital and National Taiwan University College of Medicine, Taipei, Taiwan; b Department of Medicine, National Taiwan University Cancer Center, Taipei, Taiwan; c School of Pharmacy, National Taiwan University, Taipei, Taiwan; d Department of Laboratory Medicine, National Taiwan University Hospital and National Taiwan University College of Medicine, Taipei, Taiwan; e Department of Tropical Medicine and Parasitology, National Taiwan University College of Medicine, Taipei, Taiwan; f Department of Medical Research, China Medical University Hospital, Taichung, Taiwan; g China Medical University, Taichung, Taiwan.

**Keywords:** acute cholecystitis, acute retroviral syndrome, antiretroviral therapy, case report, primary HIV infection

## Abstract

**Rationale:**

Acute retroviral syndrome is the symptomatic presentation of acute human immunodeficiency virus (HIV) infection, which often manifests as a self-limited infectious mononucleosis-like syndrome and occurs 2 to 6 weeks after exposure to HIV. Atypical manifestations including hepatitis, meningitis, or hemophagocytic lymphohistiocytosis have been reported. However, manifestations of acute acalculous cholecystitis during acute HIV infection are rarely reported.

**Patient concerns:**

A 30-year-old man with nausea and loose stools, followed by fever and abdominal pain at the right upper quadrant for 10 days.

**Diagnosis:**

Acute retroviral syndrome, complicated with acute acalculous cholecystitis.

**Interventions:**

Percutaneous transhepatic gallbladder drainage was performed and treatment with co-formulated bictegravir/emtricitabine/tenofovir alafenamide was initiated upon HIV diagnosis.

**Outcomes:**

The patient's symptoms improved after the drainage. The levels of liver enzyme including aspartate transaminase alanine aminotransferase decreased to a level within normal limits 1 month after initiation of antiretroviral therapy.

**Conclusion:**

Acalculous cholecystitis in combination with acute hepatitis could be manifestations of acute HIV infection. For individuals at risk of acquiring HIV infection who present with manifestations of acute acalculous cholecystitis, HIV testing should be considered.

## 1 Introduction

Acute human immunodeficiency virus (HIV) infection is characterized by a high concentration of HIV RNA in the plasma and rapid depletion of CD4 cell count, which often occurs 2 to 6 weeks after exposure to HIV.^[1]^ Acute HIV infection is defined as testing negative or indeterminate by Western blot in the presence of a positive p24 antigen and detectable plasma HIV RNA, and the stages of acute HIV infection are classified according to the stages defined by Fiebig et al^[2]^ Acute HIV infection often presents as a self-limited mononucleosis-like syndrome; however, atypical presentations of acute HIV infection, including acute hepatitis, aseptic meningitis, myopericarditis, and hemophagocytic lymphohistiocytosis, have been reported.^[3–6]^ Atypical presentations may lead to delayed diagnosis with a risk of onward transmission. Here, we present the clinical course of an individual presenting with acute HIV infection with manifestations mimicking acute acalculous cholecystitis.

## 2 Case presentation

**Figure 1:**
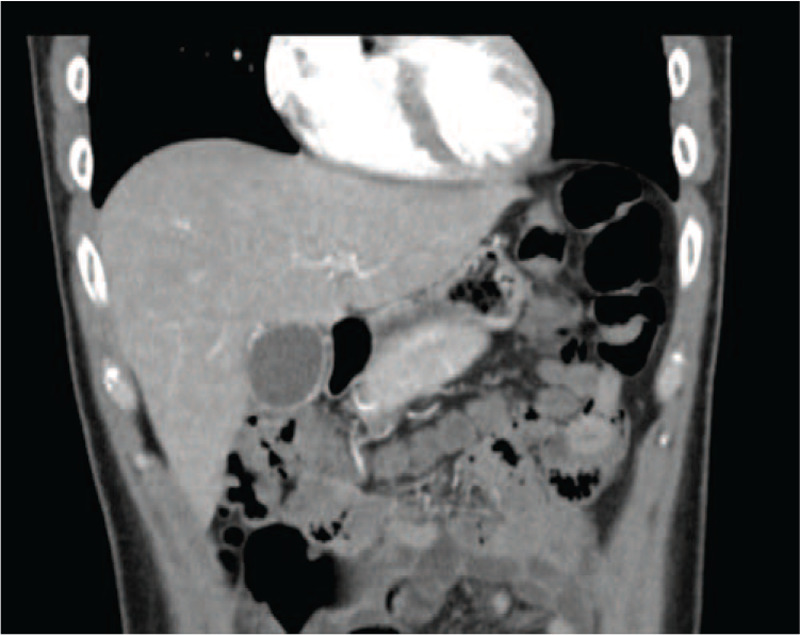
Computed tomography of the abdomen revealed a distended gallbladder with mild wall-thickening.

A 30-year-old man, previously healthy, presented with a 10-day history of nausea and passage of loose stools, followed by fevers and abdominal pain at the right upper quadrant. Upon admission, the temperature was 38.7°C, the pulse was 105 beats per minute, the blood pressure was 130/94 mmHg, the respiratory rate was 18 breaths per minute, and the oxygen saturation was 99% while the patient was breathing ambient air. Physical examination was remarkable for mildly distended abdomen and tenderness at the right upper quadrant. There was no guarding or rebound tenderness; the liver and spleen were not enlarged. The white blood cell count was 3450 cells/μL with 32% neutrophils and 51% lymphocytes; the hemoglobin level was 14.4 g/dL; the platelet count was 119,000 cells/μL; and alanine aminotransferase (ALT) was 33 U/L. Computed tomography of the abdomen revealed a distended gallbladder and mild thickening of the gallbladder wall without gallstones (Fig. 1). A diagnosis of acute acalculous cholecystitis was made and flomoxef was administered. On the fourth hospital day, a maculopapular rash developed on the abdomen and thighs. The aspartate aminotransferase (AST) and ALT level were 210 and 134 IU/L, respectively, and total and direct bilirubin levels were within normal limits. An increased level of lactate dehydrogenase (LDH) (712 IU/mL) was also noted. The platelet count decreased to 75,000 cells/μL and white blood cell count was 3090 cells/μL. Antibiotic therapy was switched to moxifloxacin because of concerns about an allergy to beta-lactams. Percutaneous transhepatic gallbladder drainage was performed because of persistent symptoms, which resulted in the resolution of the symptoms within 24 hours. However, the absence of leukocytosis and hyperbilirubinemia, which are commonly observed among patients with acute cholecystitis, prompted us to investigate the immune status of the patient and other etiologies that might cause cholecystitis.

A screening test for HIV antibodies and antigens was positive; plasma HIV RNA was >10,000,000 copies/mL and CD4 count 130 cells/μL; and Western blot was indeterminate (the presence of weak P68/66 and P25/24 bands). A diagnosis of acute HIV infection at Fiebig stage IV was made according to the results of Western blot.^[2]^ Serologic tests for acute hepatitis A, B, and C were all negative. A stool specimen examined for microscopic detection of *Cryptosporidium* spp. with the use of modified acid-fast staining was negative. He was a man who has sex with men and had unprotected sexual contact about 3 weeks before the onset of symptoms. Coformulated bictegravir, emtricitabine, and tenofovir alafenamide (BIC/FTC/TAF) were initiated on the fourth hospital day. The HIV RNA load in a drained bile specimen on the same day of BIC/FTC/TAF initiation was 2048 copies/mL. The AST and ALT levels reached the peak 1 day after the drainage (574 and 376 IU/L, respectively), while the LDH level was 1037 IU/mL. Moxifloxacin was discontinued 7 days later when blood and bile cultures showed no growth of bacteria or fungi. Fifteen days after bile drainage, he was discharged with full resolution of his symptoms on the 11th day of treatment with BIC/FTC/TAF.

During the follow-up, the levels of AST, ALT, and LDH decreased to levels within normal limits 1 month after initiation of BIC/FTC/TAF. The plasma HIV RNA had decreased to 64 copies/mL after 6 weeks of BIC/FTC/TAF, which became undetectable (<20 copies/mL) at 3 and 6 months of antiretroviral therapy. There was no relapse of his abdominal discomfort during the follow-up.

After treatment with BIC/FTC/TAF for 7 days, written informed consent was obtained from the patient for measuring the drug concentration in the blood and bile specimens. The peak and trough levels of BIC in the plasma with the use of liquid chromatography–mass spectrometry/mass spectrometry were 6430 ppb and 3809 ppb, respectively; and the BIC concentration in a 24-hour bile specimen was 3575 ppb (Supplemental Digital Content).

## 3 Discussion

Accurate diagnosis of acute HIV infection relies on prompt recognition and appropriate diagnostic testing. Flu-like symptoms during acute HIV infection, such as fever, sore throat, or lymphadenopathy, are non-specific and could be misleading.^[1,7,8]^ Moreover, atypical manifestations other than mononucleosis-like syndrome may increase the difficulty in making a timely diagnosis of acute HIV infection. Such atypical presentations may include central nervous system involvement, arrhythmia, cytopenia, and gastrointestinal symptoms.^[3]^

Cholestasis, increased bile viscosity, ischemia, and secondary infections are considered as contributing factors to the development of acute acalculous cholecystitis. Among people living with HIV, acute acalculous cholecystitis has been shown to be associated with opportunistic infections, including infections with cytomegalovirus, *Histoplasma capsulatum*, cryptosporidia, and microsporidia.^[9,10]^ Infections with hepatitis A virus, hepatitis B virus, dengue virus, and Epstein-Barr virus could cause acute acalculous cholecystitis. While HIV infection may lead to sclerosing cholangitis, acute acalculous cholecystitis associated with acute HIV infection is rarely reported.^[11,12]^

Similar to the case described by Braun et al,^[3]^ our patient with acute acalculous cholecystitis underwent percutaneous drainage with relief of the symptoms. The pathogenesis of acute acalculous cholecystitis during acute HIV infection remains unknown. In our case, HIV was detected in the bile specimen, which may be associated with endothelial damage, cholestasis, and eventually acalculous cholecystitis, similar to the case of acute acalculous cholecystitis associated with primary Epstein-Barr virus infection.^[11,13]^ Since hepatitis is a common manifestation during acute HIV infection, acute acalculous cholecystitis could also be a complication through similar pathophysiology.^[11]^

Limited data are available on the BIC concentration in body fluids other than the plasma and cerebrospinal fluid.^[14]^ While the clinical relevance is unclear, our study was the first to demonstrate the presence of a high BIC concentration in the bile specimen, which might facilitate the control of HIV replication in the gallbladder.

Our case report was limited by lacking pathologic examination of the gallbladder specimen to investigate the association between the presence of HIV RNA and the inflammation of the gallbladder. The mechanism of HIV replication leading to acute acalculous cholecystitis remains speculative. Removal of the drainage catheter when the patient recovered from acalculous cholecystitis precluded us from assessing the trend of HIV RNA load in the gallbladder.

In conclusion, our case highlights that acute acalculous cholecystitis and hepatitis could be manifestations of acute HIV infection. For individuals at risk of acquiring HIV who present with manifestations consistent with acute acalculous cholecystitis, HIV testing should be considered. Early diagnosis, bile drainage, and early initiation of antiretroviral therapy may lead to early resolution of the symptoms.

## Author contributions

**Conceptualization:** Wang-Da Liu, Chien-Ching Hung.

**Investigation:** Chih-Ning Cheng, Ya-Ting Lin, Ching-Hua Kuo, Shu-Yuan Ho.

**Methodology:** Chih-Ning Cheng, Ya-Ting Lin, Ching-Hua Kuo, Shu-Yuan Ho.

**Writing – original draft:** Wang-Da Liu.

**Writing – review & editing:** Chien-Ching Hung.

## Supplementary Material

Supplemental Digital Content

## References

[1] Lin TY Yang CJ Liu CE. Clinical features of acute human immunodeficiency virus infection in Taiwan: a multicenter study. J Microbiol Immunol Infect 2019;52:700–9. 29555411 10.1016/j.jmii.2018.01.005

[2] Fiebig EW Wright DJ Rawal BD. Dynamics of HIV viremia and antibody seroconversion in plasma donors: implications for diagnosis and staging of primary HIV infection. AIDS 2003;17:1871–9. 12960819 10.1097/00002030-200309050-00005

[3] Braun DL Kouyos RD Balmer B Grube C Weber R Günthard HF. Frequency and spectrum of unexpected clinical manifestations of primary HIV-1 Infection. Clin Infect Dis 2015;61:1013–21. 25991469 10.1093/cid/civ398

[4] Abu-Heija AA Shatta M Yeddi A Ravi AK Mutchnick M. Acute retroviral syndrome presenting as acute hepatitis. Cureus 2018;10:e3755. 30820375 10.7759/cureus.3755 PMC6388855

[5] Vandi G Calza L Girometti N. Acute onset myopericarditis as unusual presentation of primary HIV infection. Int J STD AIDS 2017;28:199–201. 27270692 10.1177/0956462416654852

[6] Manji F Wilson E Mahe E Gill J Conly J. Acute HIV infection presenting as hemophagocytic lymphohistiocytosis: case report and review of the literature. BMC Infect Dis 2017;17:633. 28931369 10.1186/s12879-017-2732-y PMC5607499

[7] Weintrob AC Giner J Menezes P. Infrequent diagnosis of primary human immunodeficiency virus infection: missed opportunities in acute care settings. Arch Intern Med 2003;163:2097–100. 14504125 10.1001/archinte.163.17.2097

[8] Schacker T Collier AC Hughes J Shea T Corey L. Clinical and epidemiologic features of primary HIV infection. Ann Intern Med 1996;125:257–64. 8678387 10.7326/0003-4819-125-4-199608150-00001

[9] Barie PS Eachempati SR. Acute acalculous cholecystitis. Gastroenterol Clin North Am 2010;39:343–57. 20478490 10.1016/j.gtc.2010.02.012

[10] Alave J Bustamante B Soto L Caceres J Seas C. Acalculous cholecystitis caused by *Histoplasma capsulatum* in a severely immunosuppressed HIV-infected patient. J Infect Dev Ctries 2011;5:235–8. 21444995 10.3855/jidc.1547

[11] Agergaard J Larsen CS. Acute acalculous cholecystitis in a patient with primary Epstein-Barr virus infection: a case report and literature review. Int J Infect Dis 2015;35:67–72. 25887813 10.1016/j.ijid.2015.04.004

[12] Imai K Misawa K Matsumura T. Progressive HIV-associated cholangiopathy in an HIV patient treated with combination antiretroviral therapy. Intern Med 2016;55:2881–4. 27725553 10.2169/internalmedicine.55.6826 PMC5088554

[13] Kaspar MB Sterling RK. Mechanisms of liver disease in patients infected with HIV. BMJ Open Gastroenterol 2017;4:e000166. 10.1136/bmjgast-2017-000166 PMC5663263 29119002

[14] Rigo-Bonnin R Tiraboschi JM Álvarez-Álvarez M. Measurement of total and unbound bictegravir concentrations in plasma and cerebrospinal fluid by UHPLC-MS/MS. J Pharm Biomed Anal 2020;185:113250. 32199329 10.1016/j.jpba.2020.113250

