# Supplementary material for: Acute HIV infection with presentations mimicking acalculous cholecystitis: A case report
Source: Medicine (Baltimore). 2021 Jul 16;100(28):e26653. doi: 10.1097/MD.0000000000026653 (PMC8284728; doi:10.1097/MD.0000000000026653)
Supplement: Supplemental Digital Content [file medi-100-e26653-s001.docx]

**Supplementary material.**

**Measurement of plasma and bile concentrations of bictegravir**

Bictegravir concentrations in plasma and bile were measured by ultra-high-performance liquid chromatography-tandem mass spectrometry (UHPLC-MS/MS). The sample preparation used a standard protein precipitation method. In brief, the samples were extracted with 100% acetonitrile for protein precipitation under the addition of bictegravir isotope (bictegravir-15N,d2) as an internal standard. The mixture was vortexed, followed by centrifugation to collect the supernatant. The supernatant was then filtered through a 0.22 μm membrane with the following 16-fold dilution by 50% acetonitrile for the UHPLC-MS/MS analysis. The analytical procedure was performed using an Agilent 1290 UHPLC system coupled with an Agilent 6470 triple quadrupole system (Agilent Technologies, Waldbronn, Germany). A ZORBAX Eclipse Plus C18 2.1×100 mm (1.8 μm) column (Agilent Technologies, Waldbronn, Germany) was used for the separation. Gradient elution was used with the mobile phase composed of a mixture of 5 mM ammonium acetate in water (solvent A) and 0.1% formic acid in acetonitrile (solvent B). The sample reservoir and the column oven were maintained at 4℃ and 25℃, respectively. The injection volume was 5 μL. The positive electrospray ionization mode was performed with the following parameters: dry gas temperature of 350℃, dry gas flow rate of 11 L min^-1^, nebulizer pressure of 50 psi, sheath gas temperature of 350℃, sheath gas flow rate of 11 L min^-1^, nozzle voltage of 0 V, capillary voltage of 3500 V. The detection transition pair was set at m/z 450.1 → 289.1 for bictegravir, and m/z 453.1 → 289.1 for bictegravir-15N,d2, respectively. The calibration curve was constructed from 20 to 3000 ng mL^−1^. The coefficient of determination for the calibration curve was larger than 0.99. The accuracy was within 100±15%, and precision was within relative standard deviation (RSD) of 15%.
